# Physical Activity Assessment—Self-Reported Versus Pedometer-Measured, and Associations with Health Markers Among University Students in South Africa

**DOI:** 10.3390/ijerph22121798

**Published:** 2025-11-27

**Authors:** Gareth Hewer, Francis Fabian Akpa-Inyang, Sizwe Vincent Mbona, Julian David Pillay, Firoza Haffejee

**Affiliations:** 1 Department of Chiropractic, Faculty of Health Sciences, Durban University of Technology, Durban 4000, South Africa; gdhewer@gmail.com; 2 Faculty of Health Sciences, Durban University of Technology, P.O. Box 1334, Durban 4000, South Africa; 3 Department of Statistics, Faculty of Applied Sciences, Durban University of Technology, Durban 4000, South Africa; sizwem@dut.ac.za; 4 Department of Basic Medical Sciences, Faculty of Health Sciences, Durban University of Technology, Durban 4000, South Africa; pillayjd@dut.ac.za (J.D.P.); firozah@dut.ac.za (F.H.)

**Keywords:** physical activity, pedometer, self-report, Sub-Saharan Africa, Sustainable Development Goals (SDG 3), university students, public health

## Abstract

Accurate measurement of physical activity is crucial for promoting health and preventing non-communicable diseases, particularly in Sub-Saharan Africa, where the dual burden of infectious and chronic diseases presents significant public health challenges. While self-reported tools are commonly used, they are often limited by recall and social desirability biases. This study aimed to compare self-reported physical activity with objectively measured pedometer data and explore their associations with fitness and anthropometric indicators among university students. A cross-sectional study was conducted among 60 full-time students at a South African university. The study was conducted in two phases: For phase 1, participants completed the International Physical Activity Questionnaire (IPAQ). For phase two, participants wore pedometers for seven consecutive days to measure total and aerobic step counts. Fitness was assessed using the Harvard Step Test to calculate the Physical Efficiency Index (PEI), and anthropometric data (BMI, waist-to-hip ratio, body fat percentage) were recorded. Statistical analyses included Spearman’s correlations, Mann–Whitney U test, Kruskal–Wallis H test, and multivariable linear regression. While 83.3% of participants self-reported high physical activity levels, pedometer data indicated that 61.7% accumulated fewer than 1500 aerobic steps per day and 53.3% recorded fewer than 5000 total steps. No significant association was found between self-reported activity and pedometer-measured steps (*p*-value = 0.748 and *p*-value = 0.835, respectively). Objective measures showed significant positive correlations between aerobic steps and PEI (*r* = 0.274, *p*-value = 0.034) and significant negative correlations with BMI (*r* = −0.489, *p*-value < 0.001) and body fat percentage (*r* = −0.255, *p*-value = 0.046). Older age and female gender were associated with lower step counts. This study reveals a significant overestimation of physical activity in self-reports compared to objective measures and stronger links between objectively measured steps and key health outcomes. The findings highlight the need to integrate objective monitoring tools into public health practice and youth-focused interventions in Sub-Saharan Africa. They underscore the importance of exploring context-specific factors influencing activity levels. Enhancing measurement accuracy is vital for advancing evidence-based strategies and achieving Sustainable Development Goal 3.

## 1. Introduction

Physical activity is increasingly recognised as a fundamental component of health and well-being, underscoring its critical role in preventing non-communicable diseases (NCDs), improving mental health, and promoting healthy ageing [1,2]. Within the framework of Sustainable Development Goal 3 (SDG 3)—“Ensure healthy lives and promote well-being for all at all ages”—regular physical activity has emerged as a key modifiable determinant of health outcomes [3,4]. This is particularly pertinent in Sub-Saharan Africa, where the dual burden of infectious diseases and rising rates of NCDs poses complex challenges for public health systems [5]. As the region undergoes rapid urbanisation and socio-economic change, there is a growing urgency to assess and promote physical activity in accurate and context-sensitive ways.

Despite its acknowledged significance, accurate quantification of physical activity remains a considerable challenge in health research [6,7]. Traditional self-reported questionnaires, while convenient and cost-effective, are often plagued by biases such as recall bias and social desirability bias, which can lead to substantial overestimations of physical activity levels [8,9]. Such inaccuracies not only hinder the validity of research findings but can also impact clinical practice, where understanding patient activity levels is vital for effective intervention planning [7]. Saint-Maurice et al., 2014, suggest that self-reported measures may inadequately capture nuanced changes in physical activity, emphasising the need for more reliable assessment methods [9]. Furthermore, studies have indicated that self-reported physical activity can differ significantly from objective measures (such as ActiGraph wGT3X-BT (Actigraph LLC, Pensacola, FL) and Personal Activity Monitor—PAM AM200 and bi-axial accelerometer—ActiTrac (IM Systems, Baltimore, MD) leading to erroneous health conclusions, especially regarding chronic diseases linked to inactivity [9,10,11,12]. Thus, objective measurement tools such as pedometers and accelerometers provide real-time data on physical movement, which are arguably more reliable for assessing physical activity [9,10,11,12].

For instance, a study utilising accelerometers revealed significant inconsistencies between self-reported and objectively measured physical activity levels [10,11]. This discrepancy holds critical implications for understanding physical activity’s relationship with health outcomes, such as cardiorespiratory fitness, body mass index (BMI), body fat percentage (BF%), and physical efficiency index (PEI) [12,13]. Additionally, demographic and anthropometric factors, including age, gender, and adiposity, may also influence both the accuracy of self-reports and actual levels of physical activity [14,15]. This means that individuals with higher adiposity may tend to underreport their weight and overreport their height [15]. Notably, Aguilar-Enríquez et al. highlight that demographic factors significantly influence motivation and engagement in physical activity; notably, their study found that more women reported engaging in physical activity than men, a trend attributed to the greater physiological demands experienced by women in adulthood compared to their male counterparts—though such gender-based interactions remain underexplored in the broader literature [16].

The growing necessity for precise physical activity assessments is further underscored by the emergence of public health crises, such as the COVID-19 pandemic, where physical activity has been markedly affected due to lockdown measures and social distancing [16]. This interruption emphasises the importance of maintaining physical activity for overall health, as evidenced by the increased risk of mental and physical health issues in sedentary individuals during times of restricted movement [17]. Furthermore, advancements in methodologies for classifying physical activity through these objective measures could bridge the gap between perceived and actual activity levels, assisting in tailoring interventions that promote healthier lifestyles [14].

The dual-method approach adopted in this study—comparing pedometer-based objective measurements with self-reported physical activity—was chosen to address longstanding concerns about the validity and reliability of self-reported data. Self-report tools such as the International Physical Activity Questionnaire (IPAQ) are widely employed due to their low cost and ease of administration; however, they are fraught with limitations including recall bias, social desirability effects, and subjective misjudgment regarding intensity and duration. These factors can significantly inflate reported activity levels [18,19]. A study by Thøgersen-Ntoumani et al. [19] illustrates how self-reports often lead to optimistic interpretations of one’s activity levels, thereby misrepresenting engagement in physical activity.

In contrast, pedometers provide a low-cost and objective method for capturing step-based activity data in real-world settings, allowing practitioners to quantify habitual physical activity with greater accuracy. This methodological triangulation, combining self-report with objective measurement, has robust theoretical support. For instance, the COM-B model (Capability, Opportunity, Motivation—Behaviour) indicates that effective behaviour change interventions must be grounded in a clear understanding of actual behaviour, which objective tools facilitate [20]. As demonstrated in research on exercise rehabilitation in adults with lung transplants, accurate behavioral assessment through objective measures elucidates the factors influencing physical activity engagement [18].

Furthermore, the Theory of Planned Behavior (TPB) elucidates the critical role of intention-behavior gaps in health behaviors, where discrepancies between perceived and actual activity highlight key barriers. This study confirms these theoretical frameworks by revealing essential insights into such gaps within the context of Sub-Saharan Africa—a region where self-reported physical activity data may be unreliable due to various socio-economic and cultural dynamics [21]. Integrating objective monitoring alongside self-perception measures enhances the assessment of physical activity behaviors and provides valuable empirical data for guiding both research and intervention strategies.

Given the noted gap in the literature surrounding self-reported physical activity in Sub-Saharan Africa, the current research aims to undertake a comprehensive analysis of physical activity assessment through systematic approaches specific to this context. It will compare self-reported physical activity levels with pedometer-derived measurements of total and aerobic step counts to critically evaluate the reliability of self-reports. Furthermore, the study will explore the associations between pedometer-measured step counts and various fitness and anthropometric measures to elucidate their indicative value for health outcomes, thereby contributing to the expanding scholarship aimed at refining physical activity assessment methods and informing targeted public health interventions in the region.

## 2. Methodology

### 2.1. Study Design and Setting

This study was conducted at a University of Technology, in Sub-Saharan Africa, from March to September 2019. It employed a cross-sectional design within a quantitative paradigm to assess and compare self-reported and objectively measured physical activity among university students. The study setting included three of the university’s main campuses.

The study was conducted in two phases. In the first phase, self-reported physical activity levels were obtained from 394 randomly selected students using the validated International Physical Activity Questionnaire (IPAQ). The second phase involved 60 students from the same cohort, who showed interest to participate in an objective assessment of physical activity using pedometers over a seven-day period. These participants undertook a fitness assessment using the Harvard Step Test and had anthropometric data recorded, including body mass index (BMI), waist-to-hip ratio (WHR), and body fat percentage (BF%).

### 2.2. Study Population

The study population comprised full-time students enrolled at a university of technology in South Africa during the 2019 academic year. Students from the Faculties of Health Sciences, Management Sciences, and Engineering and the Built Environment were randomly selected for inclusion. Only students aged 18 and above, who were free from any medical conditions that could contraindicate physical activity, were included in the study. Students were excluded if they had medical conditions that made physical activity difficult or unsafe according to the WHO guidelines [22], if they did not read or sign the informed consent form.

### 2.3. Sample Size and Participant Recruitment

The study utilised both self-reported and objective methods to assess physical activity levels, fitness, and anthropometric indicators among students. To ensure a representative and diverse sample, participant recruitment was carried out using a stratified random sampling method across the university’s three central campuses—Steve Biko, ML Sultan, and Ritson. Each campus was treated as a distinct stratum to account for geographical and faculty-related differences. Within each campus, faculties were proportionally represented, and departments were randomly selected using a random number generator. From these selected departments, eligible students were approached during scheduled lecture times and invited to participate. This approach ensured that the sample reflected variations in academic disciplines, gender, and age groups, thereby enhancing the generalizability and validity of the study findings within the broader university population: The total student population across the targeted faculties was approximately 23,500. A minimum sample size of 378 participants was calculated based on a 5% margin of error, a 95% confidence level and an assumed response distribution of 50%. With permission from the relevant departments, researchers visited selected lecture sessions to introduce the study, distribute information sheets, and obtain written consent from interested students. Those who consented were invited to complete the International Physical Activity Questionnaire (IPAQ). The selected participants completed the IPAQ was obtained and self-reported their physical activity levels. From this group, 60 students who expressed interest in participating in the second phase pedometer tracking and fitness assessments provided additional data for analysis. All data were anonymised and securely stored throughout the analysis.

In the first phase, participants (*n* = 394) completed the International Physical Activity Questionnaire (IPAQ—long form) [23], a validated self-report tool designed to assess physical activity levels (PAL) across four domains: occupational, transportation, household, and leisure-time activity [23]. The questionnaire also captured sedentary behaviour and demographic information including age, gender, race, and faculty of enrolment. Participants completed the IPAQ on-site, following the provision of a detailed information letter and signed informed consent.

**Figure 1:**
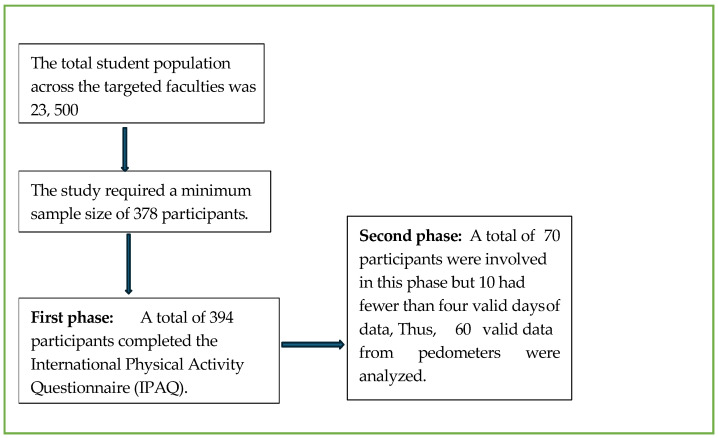
Flowchart illustrating the sample selection process in the study.

The second phase of the study included 70 participants who took part in phase 1 and who were willing to move further to phase 2; however, due to incomplete questionnaires, only 60 were analysed in this phase. This phase involved the objective assessment of physical activity and fitness using pedometers (see Figure 1). Each participant was issued an Omron Walking Style Pro 2.0—HJ-322U-E bought from HiTech Therapy, Durban, South Africa and provided with verbal and written instructions on its proper usage. Participants were instructed to wear the device continuously during waking hours for a minimum of seven consecutive days, excluding water-based activities such as bathing or swimming. To ensure compliance, participants received a daily SMS reminder to wear their pedometer, and they were required to maintain a short activity log noting any instances when the device was removed or not worn. Upon return of the device, the researcher verified the completeness of the recorded data using the device’s timestamped logs. Cases with fewer than four valid days of data were flagged as incomplete and excluded from final analyses. In instances of minor data loss, imputation was not applied; only valid, uninterrupted recordings were included to preserve data integrity.

To maintain consistency in data collection and interpretation, a single trained interviewer—who also served as the primary investigator—was responsible for distributing devices, providing instructions, conducting follow-ups, and downloading data via the pedometer’s digital interface. This approach minimized inter-interviewer variability and enhanced procedural reliability. Additionally, the pedometers were reset and tested for functionality before and after each use to prevent technical errors. These procedures ensured high compliance rates and reliable data capture for the objective assessment of participants’ physical activity levels.

In addition, participants underwent a standardised aerobic fitness assessment using the Harvard Step Test. A step bench was used for the five-minute step test, during which heart rate was monitored at 30-s intervals for five minutes using a Suunto Smart Sensor heart rate monitor bought from Suunto SA, Johannesburg, South Africa. Aerobic fitness was expressed as a Physical Efficiency Index (PEI), calculated using post-exercise heart rate recovery data.

Anthropometric data were also collected. Height was measured using a Health-O-Meter beam scale, while body mass and body fat percentage were assessed with the Omron BF511 Body Composition Monitor. Waist and hip circumferences were measured with a standard non-stretch tape, and waist-to-hip ratio (WHR) was calculated accordingly. Body mass index (BMI) was computed as weight in kilograms divided by height in meters squared (kg/m^2^).

All collected data were recorded on structured forms, anonymised using participant codes, and later entered to Microsoft Excel. The process ensured consistency and data integrity across participants and measurement tools.

### 2.4. Data Analysis

Data were imported to the Statistical Package for Social Sciences (SPSS) version 29.20 for further analysis. Summary statistics were computed to describe the distribution of all variables. The distribution of all continuous variables was first assessed using the Shapiro–Wilk test, given the small sample size (*n* = 60) [24]. The test indicated that all continuous variables were not normally distributed, as the *p*-values for all the tests were less than 0.05. Continuous variables were therefore expressed as medians with interquartile rages (IQRs), while categorical variables were summarised as frequencies and percentages.

To examine relationships between variables, Spearman’s correlation coefficients were calculated for continuous variables, such as total steps, aerobic steps, BMI, body fat percentage (BF%), and Physical Efficiency Index (PEI), using non-parametric tests. The interpretation of Spearman’s correlation coefficients was based on the following guidelines: 0–0.3 = weak, 0.3–0.6 = moderate, and 0.6–1.0 = strong, with negative values interpreted according to their magnitude [25].

A non-parametric Mann–Whitney U test was performed to assess mean differences between gender groups for selected physical and fitness outcomes. Additionally, Kruskal–Wallis H test was used to evaluate differences across self-reported physical activity levels. Furthermore, a chi-squared test was conducted to examine whether there was a significant association between two categorical variables: self-reported levels of physical activity and the categories of total and aerobic steps derived from pedometer data.

A multivariable linear regression model was used to determine the independent association of demographic (age, gender) and anthropometric variables (BMI, WHR, BF%) with the primary outcomes of interest—namely, total and aerobic steps. The model also included the Physical Efficiency Index to explore its predictive relationship with step count outcomes. For linear regression model, unstandardized beta coefficients, 95% confidence intervals (CI), t-statistics, and *p*-values were reported. Variables with *p*-values less than 0.05 were considered statistically significant.

In addition, a line graph was plotted to illustrate the mean heart rate changes over time by gender, and any statistically significant differences were examined. All step counts were aggregated per day.

## 3. Results

Table 1 presents demographics and physical activity estimates as well as the body fat estimates of a total of 60 students who participated in this study. The median (IQR) age of the study participants was 20 (19–22) years. The median total steps accumulated by participants was 4853 (3185–7358) per day, and median aerobic steps accumulated was 1175 (446–2233) per day. The median (IQR) BMI, WHR, BF% and PEI was 22.55 (20.70–25.28) $kg/m^{2}$; 76.00 (71.00–81.00); 20.90 (14.35–32.95) and 65.36 (60.30–69.93), respectively. More than half 40 (66.7%) of the participants were males. Regarding the level of physical activity, 50 (83.3%) of the participants reported high level of physical activity.

The results show that age is negatively correlated with the number of aerobic steps (r = −0.260, *p*-value = 0.043). Total steps reflect a strong positive relationship with aerobic steps (r = 0.788, *p*-value < 0.001), and therefore supporting the notion that accumulating aerobic steps is likely to result in accumulating more total steps. Physical efficiency index was also found to have a statistically significant, albeit weak positive relationship with aerobic steps (r = 0.274, *p*-value = 0.034), which suggests that aerobic steps positively influence PEI. Furthermore, BMI had a significant negative moderate correlation with aerobic steps (r = −0.489, *p*-value < 0.001), implying that the accumulation of aerobic steps negatively impacts BMI. Similarly, BF% was found to be negatively correlated with the aerobic steps (r = −0.255, *p*-value = 0.046).

**Figure 2:**
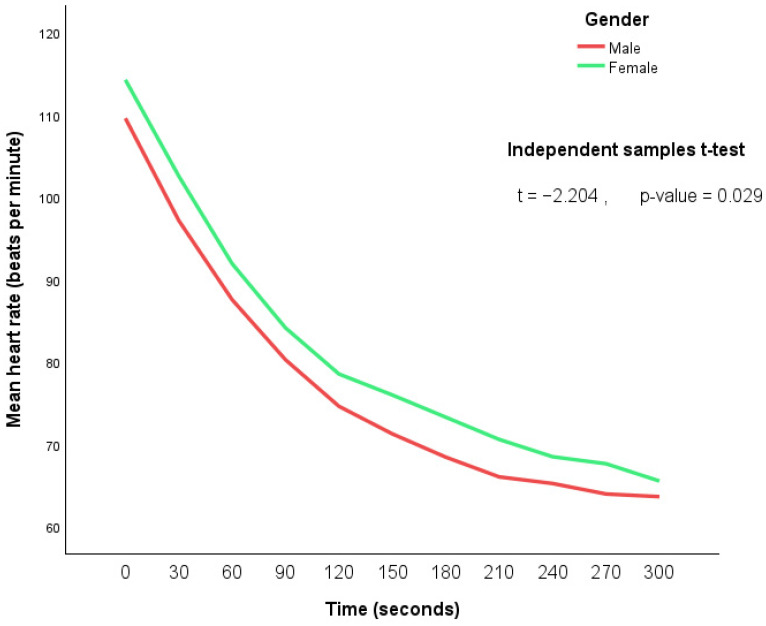
Relationship between gender and mean heart rate.

The heart rate measured at 30-s intervals was found to be significantly higher for female participants than male participants (*p*-value = 0.029) for the Harvard Step Test. These associations may reflect autonomic regulation or motivational factors, but further research is needed. The graph in Figure 2 further shows a consistent decrease in mean heart rate in both males and females during the observation period. Further investigation could help clarify the underlying factors contributing to this disparity.

The results presented in Table 1 indicate that 50 (83.3%) of participants reported a high level of physical activity. However, when compared to the pedometer-measured steps (Table 2), 37 (61.7%) of these participants recorded fewer than 1500 aerobic steps, and 32 (53.3%) recorded fewer than 5000 total steps. Thus, statistical analysis revealed no significant relationship between the self-reported level of physical activity and the pedometer-measured number of aerobic steps (*p*-value = 0.748) or total steps (*p*-value = 0.835). This suggests that most participants who reported a high level of physical activity in the questionnaire did not exhibit correspondingly high aerobic or total step counts as recorded by the pedometer. The results presented in Table 2 also show that the majority of participants, regardless of gender or race, reported high levels of physical activity. However, the associations between self-reported physical activity levels and both gender and race were not statistically significant (*p*-value > 0.05).

The results presented in Table 3 (Panel A) show that the older the student, the lower the chances of taking more total steps (β = −0.09; 95% CI: −0.15 to −0.03). Additionally, the number of total steps increased with higher Physical Exercise Index (PEI) (β = 0.10; 95% CI: 0.01 to 0.19) and decreased with higher Body Mass Index (BMI) (β = −0.11; 95% CI: −0.15 to −0.07). Variables such as Waist-to-Hip Ratio (WHR) and Body Fat Percentage (BF%) were not significantly associated with the number of self-reported steps (*p*-value > 0.05), indicating no measurable impact (positive or negative) on total step counts. Furthermore, there was no significant difference in the number of total steps based on gender or self-reported levels of physical activity (*p*-value = 0.350).

As presented in Table 3 (Panel B), the results reveal that the age of the respondent is significantly associated with the low likelihood of the number of aerobic steps taken (β = −0.11; 95% CI: −0.15 to −0.07). This implies that the older the participant, the less aerobic steps are accumulated. With regard to performing total steps, the finding suggests a strong positive relationship between total steps and aerobic steps. For each unit increase in total steps, the aerobic steps increase by a factor of 1.27 (β = 1.27; 95% CI: 1.21 to 1.33). The results show that aerobic steps significantly increased with a decrease in BMI and BF% (β = −0.15; 95% CI: −0.23 to −0.07) and (β = −0.10; 95% CI: −0.12 to −0.08), respectively. This means that participants with low BMI and BF% accumulated more aerobic steps. Female participants accumulated, on average, 0.17 fewer aerobic steps than male participants (*β* = −0.17; 95% CI: −0.29 to −0.05).

## 4. Discussion

Physical activity is universally recognised as a critical determinant of health and well-being, with substantial evidence linking regular movement to improved cardiovascular function, reduced adiposity, enhanced mental health, and lower risk of non-communicable diseases (NCDs) [1,2]. Its role in maintaining a healthy body composition, promoting metabolic efficiency, and improving cardiorespiratory fitness has been well-documented across diverse populations [12,13]. In the context of university students, physical activity not only supports physiological development but also contributes to better academic performance and mental resilience. Objective health indicators such as body mass index (BMI), body fat percentage (BF%), and the Physical Efficiency Index (PEI) are commonly used to quantify the physiological impact of physical activity [26,27]. This study reinforces these relationships by demonstrating significant associations between pedometer-measured aerobic steps and key health markers, including inverse relationships with BMI and BF%, and a positive association with PEI.

However, these relationships are not uniform across populations and are often influenced by a range of demographic and psychosocial factors. Gender, in particular, plays a significant role in shaping physical activity behaviours, perceptions, and outcomes. In this study, female participants recorded significantly lower levels of objectively measured aerobic steps and heart rate responses compared to males, suggesting both lower engagement in sustained physical activity and potentially different fitness profiles. These findings echo prior research indicating that women, especially in adulthood, may face unique physiological, sociocultural, and motivational barriers to physical activity [28,29]. Factors such as safety concerns, body image perceptions, time constraints, and social expectations can differentially influence women’s participation in physical activity, thereby moderating the relationship between activity levels and health outcomes [28,29]. Understanding these gendered patterns is critical for developing targeted interventions and inclusive health promotion strategies, especially within educational institutions and youth-focused settings.

Gender differences were evident: female participants demonstrated significantly higher heart rates and fewer aerobic steps, on average, compared to their male counterparts. These observed differences can be partly explained by sociocultural factors, biological variations in activity preferences, and differing perceptions of physical exertion [30]. The literature reflects these discrepancies, as several studies highlight gender-based preferences in physical activity engagement levels, indicating a need for targeted health interventions that take these factors into account [16,31].

The results revealed significant divergences that resonate with the broader public health challenges highlighted within the Sustainable Development Goals (SDGs) framework, particularly SDG 3, which emphasises good health and well-being. The discrepancies identified in this research may serve to highlight the critical public health issues affecting this region, where reliable measurements of physical activity are vital for tailored interventions and health promotion strategies.

Despite a high level of self-reported physical activity, pedometer data revealed a stark contrast, with a significant proportion falling short of even modest physical activity recommendations. Specifically, 37 (61.7%) participants recorded fewer than 1500 aerobic steps per day, and 32 (53.3%) participants accumulated fewer than 5000 total steps, confirming a common outcome in physical activity measurement research where self-reports tend to overestimate actual activity levels [6,10].

These discrepancies align with findings in previous studies, such as those by Ghaneapur et al., as well as Shenoy and Jahan, who conducted a study on the comparison and relationship of two methods of measuring physical activity and its related components among middle age adults and women: pedometer versus self-report [31,32]. They found a trend where self-reported physical activity often did not correlate well with objective measures, reinforcing results that suggest reliance on self-reports could lead to misclassification of individuals’ activity levels and associated health risks [31,32]. The absence of significant associations between self-reported physical activity and both aerobic and total step counts emphasises the limitations of subjective assessments in detecting true activity patterns [10,31].

In addition, the study found that pedometer-measured activity exhibited robust associations with several key health indicators. The significant positive relationship between aerobic step counts and the Physical Efficiency Index (PEI) suggests that higher volumes of aerobic activity may contribute to enhanced cardiorespiratory fitness, corroborating prior research linking increased physical activity with improved fitness outcomes [26,27]. Furthermore, the inverse relationships observed between aerobic step counts and both BMI and body fat percentage (BF%) are consistent with the literature supporting the connection between physical activity and favourable body composition outcomes [31,32]. Specifically, higher aerobic activities were negatively correlated with BMI, aligning with established evidence that suggests importance of physical activity in mitigating obesity and promoting healthier body compositions in vulnerable populations across Sub-Saharan Africa [10,33].

Demographic factors, including age and gender, emerged as significant predictors of physical activity patterns, reflecting patterns seen in the broader literature. The study observed an inverse association between age and both total and aerobic steps, indicating a potential decline in physical activity levels as students mature. Regression analyses indicated that each additional year of age was linked to a decreased likelihood of engaging in aerobic stepping and total steps, findings mirrored in prior studies that attribute such declines to increased academic demands and shifts in lifestyle or motivation as students progress through their academic careers [28,29].

Further, multivariable regression analyses showed that higher PEI significantly enhanced the likelihood of achieving both total and aerobic step counts, while increased BMI consistently decreased those chances. This reinforces the idea that fitness, as measured by PEI, plays a critical role in facilitating higher engagement in physical activity. However, the lack of significant associations found between waist-to-hip ratio (WHR) or BF% with total step counts suggests these anthropometric indicators may be less sensitive to general walking activities and might be better correlated with more intense forms of physical exertion [34,35].

One of the key strengths of this study lies in its dual-method approach, directly comparing self-reported physical activity with objectively measured pedometer data among university students in a Sub-Saharan African context. While numerous studies have investigated this discrepancy in high-income countries, this research provides novel insight into these dynamics within a low- to middle-income setting, where such data remains scarce [7,11,31,32]. Furthermore, by linking both self-reported and objectively measured activity to anthropometric and fitness indicators such as BMI, body fat percentage (BF%), and the Physical Efficiency Index (PEI), the study offers a comprehensive view of how perceived and actual physical activity correlate with important health markers in young adults. This work addresses a critical gap in the literature and contributes evidence toward refining physical activity surveillance strategies tailored to the unique demographic and epidemiological transitions occurring in Sub-Saharan Africa. The inclusion of objective heart rate monitoring during fitness testing further enhances the physiological validity of the data collected. Moreover, the study’s timing, situated post-COVID-19 restrictions, provides a relevant snapshot of physical activity behaviours in an evolving public health environment where mobility patterns and lifestyle habits have shifted significantly.

A few limitations must be acknowledged: The relatively small sample size (*n* = 60) for the pedometer-measured phase, while adequately powered for the study’s aims, may limit the generalisability of the findings to broader student populations or to other age groups. Additionally, while pedometers offer a practical and accessible means of capturing step counts, they do not capture upper body movements, swimming, or intensity variations beyond step speed, potentially underestimating total physical activity. Finally, despite efforts to ensure compliance, the accuracy of pedometer data relies on participant adherence to wearing protocols, which could introduce measurement variability. It is also worth noting that one of the limitations of the study is that the quantification of PAL conducted via questionnaire (IPAQ) and pedometer (steps) were not done concurrently in the same day, this may be one of the reasons the PAL was found to be significantly overestimated. Notwithstanding these limitations, this study offers important and novel contributions by highlighting discrepancies between perceived and actual physical activity in a critical demographic, providing actionable evidence for health promotion strategies aimed at achieving Sustainable Development Goal 3 in Sub-Saharan Africa.

Overall, the collective results of this study advocate for the use of objective measurement tools, such as pedometers, to obtain reliable insights into real-world physical activity patterns. While self-reports can serve to gauge perceptions or attitudes toward physical activity, the findings clearly indicate that objective measures are necessary for accurately determining actual activity levels and their physiological consequences [36,37]. This highlights an urgent need for a paradigm shift in physical activity assessment practices in both research and public health settings.

### 4.1. Theoretical and Conceptual Advancement

This study contributes to the theoretical and conceptual advancement of physical activity research in several key ways. First, by examining self-reported and objectively measured physical activity within a Sub-Saharan African university population, it challenges the widespread reliance on subjective instruments like the International Physical Activity Questionnaire (IPAQ), which is one of the most widely used self-report tools for estimating PAL in population surveys. Conceptually, the study extends understanding of how misperceptions of physical activity, particularly among youth can obscure meaningful relationships with critical health indicators such as BMI, body fat percentage, and aerobic fitness. The study also foregrounds the influence of demographic variables such as age and gender on actual versus perceived activity, offering a nuanced framework for understanding disparities in health behaviours. The integration of pedometer data with fitness and anthropometric outcomes strengthens the case for a multidimensional model of physical activity assessment that is both context-specific and biologically grounded. Importantly, by aligning its findings with Sustainable Development Goal 3, the study introduces a public health logic to activity surveillance in African contexts, thereby advocating for more evidence-informed, technology-integrated interventions. Collectively, these contributions pave the way for reconceptualising physical activity not merely as a self-reported behaviour but as a quantifiable, health-linked practice requiring methodological rigour and contextual sensitivity.

### 4.2. Implications

The implications of this study extend to various facets of public health practice and policy, particularly in the context of SDG 3 and the wider public health challenges facing Sub-Saharan Africa. First, the reliance on self-reported physical activity without corroboration from objective measures could lead to misclassification and an underestimation of inactivity-related health risks [10,33]. Interventions aimed at promoting physical activity should consider integrating objective monitoring tools to enhance measurement accuracy and enable tailored health recommendations. Furthermore, health promotion efforts directed toward university students, particularly among older cohorts and female participants, should address specific barriers to physical activity involvement and promote sustained, aerobic forms of exercise that align with evidence-based health guidelines. By addressing these disparities and refining assessment strategies, we can contribute to more effective public health initiatives aimed at improving health outcomes among young adults and across various demographics.

### 4.3. Policy Implications and Local Application

The findings of this study carry important policy implications for health promotion and disease prevention strategies within South Africa and similar sub-Saharan African contexts. The substantial discrepancy between self-reported and objectively measured physical activity underscores the urgent need for policy frameworks that incorporate objective monitoring tools such as pedometers and wearable technologies into routine health assessments, particularly within schools and higher education institutions. Integrating such tools can enhance the reliability of physical activity surveillance and inform more accurate risk stratification and interventions. Locally, universities and provincial departments of health can draw on these findings to develop contextually relevant physical activity guidelines, gender-sensitive wellness programs, and campus-wide fitness initiatives that address demographic disparities in physical activity engagement. Moreover, the evidence presented here supports the inclusion of physical activity tracking in student health policies, reinforcing the role of educational institutions as strategic settings for advancing Sustainable Development Goal 3. By translating research into action at the institutional and policy level, this study offers a roadmap for strengthening health systems, promoting active lifestyles, and reducing the long-term burden of non-communicable diseases across South Africa.

### 4.4. Recommendations

This study highlights the need to integrate objective physical activity monitoring, such as pedometers, into public health surveillance and university-based interventions in Sub-Saharan Africa. Objective tools help address the well-documented limitations of self-reported data, including recall and social desirability bias. Given the observed disparities by age and gender, targeted and inclusive programs are essential—particularly for female and older students. Universities should embed physical activity promotion into campus life through structured classes and wellness initiatives, while also encouraging the use of wearable technology for accurate self-monitoring. Future research should adopt mixed-methods approaches to explore behavioural and contextual factors, supporting the development of more effective, evidence-based interventions aligned with Sustainable Development Goal 3.

## 5. Conclusions

This study reveals a clear disconnect between self-reported and objectively measured physical activity among university students, with 83.3% reporting high activity levels, yet pedometer data showing that 62% did not reach the recommended 1500 aerobic steps and 64% failed to meet 5000 total steps per day. Objective step counts were significantly associated with key health indicators—positively with the Physical Efficiency Index, and negatively with BMI and body fat percentage—while self-reported data showed no such associations. These findings reaffirm concerns over the validity of self-reported physical activity and highlight the value of integrating objective monitoring tools in both research and practice.

The results underscore the need for a shift in public health strategies toward technology-supported, evidence-based assessments that accurately reflect activity patterns. By adopting objective measures like pedometers in schools and universities, health promotion efforts can more effectively identify and support at-risk groups, particularly women and older students. Ultimately, enhancing physical activity surveillance through reliable tools will strengthen the design of targeted interventions and advance Sustainable Development Goal 3 in Sub-Saharan Africa.

### Ethics Approval

Ethical approval to conduct the research was obtained from the Institutional Research Ethics Committee (IREC) at the Durban University of Technology (ethical clearance number: IREC 038/18; Date; 27 July 2018). Prior to data collection, all participants were provided with a detailed letter of information and were required to sign informed consent forms. Participation in the study was entirely voluntary, with assurances of confidentiality, anonymity, and the right to withdraw at any time without penalty.

## Figures and Tables

**Table 1:** Descriptive statistics of study participants (*n* = 60).

Variable	Median (IQR)	*n* (%)	Statistics (*p*-Value)
Age in years	20 (19–22)	-	−0.260 (0.043) ^a^
Gender	Male	40 (66.7)	2.134 (0.035) ^b^
	Female	20 (33.3)	
Race	Black/African	49 (81.7)	3.686 (0.025) ^c^
	Indian	7 (11.7)	
	White	4 (6.7)	
Level of physical activity (self-reported)	Low	2 (3.3)	2.041 (0.115) ^c^
	Moderate	8 (13.3)	
	High	50 (83.3)	
Total steps/day	4853 (3185–7358)	-	0.788 (<0.001) ^a^
Aerobic steps/day	1175 (446–2233)	-	1
BMI	22.55 (20.70–25.28)	-	−0.489 (<0.001) ^a^
WHR	76.00 (71.00–81.00)	-	−0.151 (0.250) ^a^
BF%	20.90 (14.35–32.95)	-	−0.255 (0.046) ^a^
PEI	65.36 (60.30–69.93)	-	0.274 (0.034) ^a^

*n* = number; % = percentage; IQR = interquartile range; BMI = Body Mass Index; WHR = Waist-to-Hip Ratio; BF% = Body Fat Percentage; PEI = Physical Efficiency Index; a = Spearman’s correlation analysis; b = Mann–Whitney U test; c = Kruskal–Wallis H test.

**Table 2:** Distribution of self-reported physical activity levels across selected variables.

		Self-Reported Levels of Physical Activity			TOTAL	*p*-Value
		Low	Moderate	High		
Aerobic steps	<1500	1	5	31	37	0.748
	1500–3500	1	1	12	14	
	>3500	0	2	7	9	
Total steps	<5000	1	4	27	32	0.835
	5000–9999	1	4	18	23	
	>9999	0	0	5	5	
Gender	Male	1	3	36	40	0.139
	Female	1	5	14	20	
Race	Black/African	2	6	41	49	0.645
	Indian	-	2	5	7	
	White	-	-	4	4	

**Table 3:** Multivariable linear regression model.

Model	β	Standard Error	95% CI	t-Statistic	*p*-Value
Panel A: Total steps					
Age	−0.09	0.03	−0.15 to −0.03	−3.00	0.004
PEI	0.10	0.05	0.01 to 0.19	2.00	0.045
BMI	−0.11	0.02	−0.15 to −0.07	−5.50	<0.001
WHR	−0.18	0.14	−0.45 to 0.09	−1.29	0.247
BF%	−0.07	0.06	−0.19 to 0.05	−1.17	0.243
	Gender				
Male (Ref)	-	-	-	-	-
Female	−0.12	0.19	−0.50 to 0.26	−0.63	0.530
	Level of physical activity				
Low (Ref)	-	-	-	-	-
Moderate	0.11	0.35	−0.59 to 0.81	0.31	0.755
High	0.15	0.22	−0.29 to 0.59	0.68	0.498
Panel B: Aerobic steps					
Age	−0.11	0.02	−0.15 to −0.07	−5.50	<0.001
Total steps	1.27	0.03	1.21 to 1.33	42.33	<0.001
PEI	0.10	0.01	0.08 to 0.12	10.00	<0.001
BMI	−0.15	0.04	−0.23 to −0.07	−3.75	0.001
WHR	−0.22	0.12	−0.46 to 0.02	−1.83	0.071
BF%	−0.10	0.01	−0.12 to −0.08	−10.00	<0.001
	Gender				
Male (Ref)	-	-	-	-	-
Female	−0.17	0.06	−0.29 to −0.05	−2.77	0.006
	Level of physical activity				
Low (Ref)	-	-	-	-	-
Moderate	0.12	0.33	−0.54 to 0.78	0.36	0.717
High	0.39	0.18	0.03 to 0.75	2.17	0.031

*β* = Unstandardised Coefficient; CI = confident interval; Ref = reference category; BMI = Body Mass Index; WHR = Waist-to-Hip Ratio; BF% = Body Fat Percentage; PEI = Physical Efficiency Index.

## Data Availability

The raw data supporting the conclusions of this article will be made available by the authors on request.

## References

[1] Saqib Z.A., Dai J., Menhas R., Mahmood S., Karim M., Sang X., Weng Y. (2020). Physical Activity is a Medicine for Non-Communicable Diseases: A Survey Study Regarding the Perception of Physical Activity Impact on Health Wellbeing. Risk Manag. Healthc. Policy.

[2] Budreviciute A., Damiati S., Sabir D.K., Onder K., Schuller-Goetzburg P., Plakys G., Katileviciute A., Khoja S., Kodzius R. (2020). Management and Prevention Strategies for Non-communicable Diseases (NCDs) and Their Risk Factors. Front. Public Health.

[3] Guégan J.F., Suzán G., Kati-Coulibaly S., Bonpamgue D.N., Moatti J.-P. (2018). Sustainable Development Goal #3, “health and well-being”, and the need for more integrative thinking. Vet. México OA.

[4] Dai J., Menhas R. (2020). Sustainable Development Goals, Sports and Physical Activity: The Localization of Health-Related Sustainable Development Goals Through Sports in China: A Narrative Review. Risk Manag. Healthc. Policy.

[5] Kassa A.A., Zewdie S., Taddele M. (2024). Noncommunicable disease behavioral risk factors in Sub Saharan Africa: A protocol of systematic review and meta-analysis. PLoS ONE.

[6] Onagbiye S.O., Bester P. (2022). Physical Inactivity as a Wicked Problem in Sub-Sahara Africa: Overview and Recommendations. Open Public Health J..

[7] Steultjens M., Bell K., Hendry G. (2023). The challenges of measuring physical activity and sedentary behaviour in people with rheumatoid arthritis. Rheumatol. Adv. Pract..

[8] Caputo A. (2017). Social desirability bias in self-reported well-being measures: Evidence from an online survey. Univ. Psychol..

[9] Saint-Maurice P.F., Welk G.J., Beyler N.K., Bartee R.T., Heelan K.A. (2014). Calibration of self-report tools for physical activity research: The Physical Activity Questionnaire (PAQ). BMC Public Health.

[10] Monyeki M.A., Moss S.J., Kemper H.C.G., Twisk J.W.R. (2018). Self-Reported Physical Activity is Not a Valid Method for Measuring Physical Activity in 15-Year-Old South African Boys and Girls. Children.

[11] Quinlan C., Rattray B., Pryor D., Northey J.M., Anstey K.J., Butterworth P., Cherbuin N. (2021). The accuracy of self-reported physical activity questionnaires varies with sex and body mass index. PLoS ONE.

[12] Hills A.P., Mokhtar N., Byrne N.M. (2014). Assessment of Physical Activity and Energy Expenditure: An Overview of Objective Measures. Front. Nutr..

[13] Purković M., Vukašinović D., Radak U., Maksimović M. (2021). The importance of physical activity in diabetes. Biomed. Istraživanja.

[14] Mamiya H., Fuller D. (2023). Quantifying the Uncertainty of Human Activity Recognition Using a Bayesian Machine Learning Method: A Prediction Study. medRxiv.

[15] Mardini M.T., Bai C., Wanigatunga A.A., Saldana S., Casanova R., Manini T.M. (2021). Age differences in estimating physical activity by wrist accelerometry using machine learning. Sensors.

[16] Aguilar-Enríquez R.I., Rivera A., Flores-Chico B., de la Rosa L.E.L., Fernández-Montiel Y.L. (2021). The importance of physical activity in COVID-19 times. World J. Adv. Res. Rev..

[17] Brady S.M., Fenton S.A., Metsios G.S., Bosworth A., Duda J.L., Kitas G.D., Veldhuijzen van Zanten J.J. (2021). Different types of physical activity are positively associated with indicators of mental health and psychological wellbeing in rheumatoid arthritis during COVID-19. Rheumatol. Int..

[18] Yang H., Liu S., Chen J., Qiao Y., Wang C., Zhang W., Wei L., Chen R. (2024). Perceptions of barriers to and facilitators of exercise rehabilitation in adults with lung transplantation: A qualitative study in China. BMC Pulm. Med..

[19] Thøgersen-Ntoumani C., Kritz M., Grunseit A., Chau J., Ahmadi M., Holtermann A., Koster A., Tudor-Locke C., Johnson N., Sherrington C. (2023). Barriers and enablers of vigorous intermittent lifestyle physical activity (VILPA) in physically inactive adults: A focus group study. Int. J. Behav. Nutr. Phys. Act..

[20] Muhwava L.S., Murphy K., Zarowsky C., Levitt N. (2019). Experiences of lifestyle change among women with gestational diabetes mellitus (GDM): A behavioural diagnosis using the COM-B model in a low-income setting. PLoS ONE.

[21] Flannery C., McHugh S., Anaba A.E., Clifford E., O’Riordan M., Kenny L.C., McAuliffe F.M., Kearney P.M., Byrne M. (2018). Enablers and barriers to physical activity in overweight and obese pregnant women: An analysis informed by the theoretical domains framework and COM-B model. BMC Pregnancy Childbirth.

[22] Bull F.C., Al-Ansari S.S., Biddle S., Borodulin K., Buman M.P., Cardon G., Carty C., Chaput J.P., Chastin S., Chou R. (2020). World Health Organization 2020 guidelines on physical activity and sedentary behaviour. Br. J. Sports Med..

[23] Craig C.L., Marshall A.L., Sjöström M., Bauman A.E., Booth M.L., Ainsworth B.E., Pratt M., Ekelund U., Yngve A., Sallis J.F. (2003). International physical activity questionnaire: 12-country reliability and validity. Med. Sci. Sports Exerc..

[24] Shapiro S.S., Wilk M.B. (1965). An Analysis of Variance Test for Normality (Complete Samples). Biometrika.

[25] Dancey C.P., Reidy J. (2007). Statistics Without Maths for Psychology.

[26] Kim D.H., Cho Y.H., Seo T.B. (2022). Correlation between physical efficiency index using Harvard step test and heart rate variation in college students. J. Exerc. Rehabil..

[27] Wang Q., Qian J., Pan H., Ju Q. (2023). Relationship between body composition and upper limb physical fitness among Chinese students: 4-Year longitudinal follow-up and experimental study. Front. Physiol..

[28] Huang G., Chen Y., Lee B., Qiu Y., Mao A., Liang M., Liu M. (2025). A study on the effects of modified sprint interval trainingon physical fitness test scores and the quantitative and dose-response relationships among Chinese male university students. Front. Physiol..

[29] Suryadinata R.V., Wirjatmadi B., Adriani M., Lorensia A. (2020). Effect of age and weight on physical activity. J. Public Health Res..

[30] Rascon J., Trujillo E., Morales-AcuÑa F., Gurovich A.N. (2020). Differences between Males and Females in Determining Exercise Intensity. Int. J. Exerc. Sci..

[31] Ghaneapur M., Ezati Asar M., Belji Kangarlou M., Saleh E. (2025). Comparison of two methods of measuring physical activity and its related components among middle age women: Pedometer versus self-report. PLoS ONE.

[32] Jahan N., Shenoy S. (2017). Relation of pedometer steps count & self reported physical activity with health indices in middle aged adults. Diabetes Metab. Syndr. Clin. Res. Rev..

[33] Muti M., Ware L.J., Micklesfield L.K., Ramsay M., Agongo G., Boua P.R., Kisiangani I., Cook I., Gómez-Olivé F.X., Crowther N.J. (2023). Physical Activity and Its Association with Body Mass Index: A Cross-Sectional Analysis in Middle-Aged Adults From 4 Sub-Saharan African Countries. J. Phys. Act. Health.

[34] Myers J., Kokkinos P., Nyelin E. (2019). Physical Activity, Cardiorespiratory Fitness, and the Metabolic Syndrome. Nutrients.

[35] Espinoza Gutierrez G.A., Yance-Cacñahuaray G., Runzer-Colmenares F.M., Chambergo-Michilot D., Falvy-Bockos I., Vidal-Neira L.F. (2022). Association Between Hip-Waist Ratio and Physical Performance in Older Adults. Electron. J. Gen. Med..

[36] Carson V., Lee E.Y., Hewitt L., Jennings C., Hunter S., Kuzik N., Stearns J.A., Unrau S.P., Poitras V.J., Gray C. (2017). Systematic review of the relationships between physical activity and health indicators in the early years (0–4 years). BMC Public Health.

[37] Poitras V.J., Gray C.E., Borghese M.M., Carson V., Chaput J.P., Janssen I., Katzmarzyk P.T., Pate R.R., Connor Gorber S., Kho M.E. (2016). Systematic review of the relationships between objectively measured physical activity and health indicators in school-aged children and youth. Appl. Physiol. Nutr. Metab..

